# Does hand dominance influence clinical outcomes and implant survival in shoulder arthroplasty?

**DOI:** 10.1016/j.jor.2024.10.052

**Published:** 2024-11-02

**Authors:** Pit Hetto, Matthias Wolf, Stefanos Tsitlakidis, Julian Deisenhofer, Thomas Bruckner, David Spranz, Paul Mick

**Affiliations:** a Department of Orthopedic and Trauma Surgery, University of Heidelberg, Schlierbacher Landstraße 200a, 69118, Heidelberg, Germany; b Institute of Medical Biometry and Informatics, University of Heidelberg, Im Neuenheimer Feld 305, 69120, Heidelberg, Germany

**Keywords:** TSA, Osteoarthritis, Outcome, Satisfaction, Hand dominance

## Abstract

**Background:**

Total shoulder arthroplasty (TSA) is a successful treatment method for patients with end-stage glenohumeral osteoarthritis and different factors influencing the clinical outcome have been determined. However, the role of hand dominance on the postoperative clinical results and implant survival is not well analyzed. Hypothesis: Hand Dominance does not influence the outcome after TSA.

**Patients and methods:**

In this single center cohort study, 138 consecutive patients with primary Osteoarthritis (OA)were treated with a TSA and evaluated with a mean follow-up of 45 months (range 24–158 months). 62.32 % (86 patients) underwent TSA on the dominant side (DOM group) and 37.68 % (52 patients) on their non-dominant side (NON-DOM group). For clinical evaluation the adjusted Constant-Murley (CS) score, pain (Numeric analog scale (NAS)), subjective satisfaction and range of motion (ROM) were recorded. Furthermore, survivorship analysis was performed for the endpoint revision for any reason.

**Results:**

Both dominant (DOM) and non-dominant (NON-DOM) groups demonstrated significant improvement in all clinical outcomes from pre-to postoperative (p < 0.0001) with no between-group differences (p > 0.05). Implant survival was 98.8 % at 123 months (DOM) and 90 % at 158 months (NON-DOM) (p = 0.89).

**Conclusion:**

Hand dominance did not influence postoperative outcomes, survivorship, or age at time of surgery. Patients can expect excellent results after TSA regardless of their predominant dexterity.

**Level of evidence:**

Retrospective cohort study, Level III.

## 1 Introduction

Total shoulder arthroplasty (TSA) has been shown to yield favorable medium-to long-term clinical outcomes and serves as an effective intervention for patients with advanced glenohumeral osteoarthritis.^1^, ^2^, ^3^, ^4^, ^5^ Nevertheless, not all patients achieve uniform improvements following TSA, with variability reported in subjective satisfaction, pain relief, and functional recovery.^1^^,^^6^^,^^7^ Several factors influencing clinical outcomes have been reported, including the severity of preoperative osteoarthritis, rotator cuff integrity, and hand dominance.^6^^,^^8^^,^^9^

In contrast, studies of total knee arthroplasty (TKA) has demonstrated poorer postoperative outcomes in cases with less severe preoperative osteoarthritis, prompting some authors to advocate for TKA only in advanced stages of the disease.^10^, ^11^, ^12^, ^13^ However, these findings from TKA may not be directly applicable to the shoulder joint, which is non-weight-bearing and depends more on dynamic muscular stabilization. Sowa et al. reported that milder osteoarthritis did not adversely affect postoperative shoulder function or patient satisfaction following TSA.^9^

The role of hand dominance in TSA outcomes and implant longevity remains inconclusive. Whether hand dominance influences arthroplasty survival or postoperative shoulder function has yet to be clearly determined. Cvetanovich et al. found that patients who underwent TSA on their dominant arm exhibited superior postoperative range of motion, though no significant differences were observed in pain levels or patient-reported outcomes at a mean follow-up of 23 months.^14^ Collin et al. identified a relationship between hand dominance and the progression of radiolucent lines on imaging studies.^15^ However, multiple studies have reported no significant association between hand dominance and TSA outcomes.^16^, ^17^, ^18^, ^19^ In the setting of shoulder hemiarthroplasty for fracture, Le Blanc et al. observed notably worse outcomes in the dominant arm.^20^

## 2 Materials and methods

Between November 2002 and March 2009, 175 consecutive patients underwent total shoulder arthroplasty (TSA) in our department and were included in a prospective database. The diagnosis was primary osteoarthritis in all cases, with the same anatomical prosthesis implant utilized (Aequalis Total Shoulder; Wright Medical®, Memphis, TN, USA). Exclusion criteria were preoperative rotator cuff tears, prior shoulder surgery, and neurological impairment of the treated shoulder. Informed consent was obtained from all study participants. Minimum follow-up of 24 months was required for inclusion. Mean patient age at the time of surgery was 68.7 years (range 51–89 years), mean follow-up was 45 months (range 24–158 months).

### 2.1 Operative technique and implants

The indication for shoulder arthroplasty in all cases was glenohumeral osteoarthritis, and a deltopectoral approach was utilized for each procedure. The surgical technique for total shoulder arthroplasty, as used in this study, has been previously detailed.^21^ A consistent hybrid fixation, third-generation anatomic stemmed humeral component was implanted in all cases (Aequalis Total Shoulder; Wright Medical®, Memphis, TN, USA). In addition, every procedure involved the use of a uniform peripheral-pegged, convex-backed, third-generation glenoid component (Aequalis Total Shoulder; Wright Medical®, Memphis, TN, USA). Standard polymethyl methacrylate bone cement (Biomet, Warsaw, USA) was employed for the cement fixation of both the humeral and glenoid implants.

### 2.2 Clinical evaluation

All patients were evaluated using the Constant–Murley scoring system (CS), with age- and sex-related adjustments according to Yian et al..*^22^* The range of motion for active motion of the shoulder was analyzed for flexion in the sagittal plane, abduction in the coronal plane, and external rotation with the arm hanging at the side and with elbow flexion of 90°.

Pain, activity, mobility, and strength were classified using the Constant–Murley scoring system: pain: 0 points (severe pain) to 15 points (no pain); activity: 0 points (no activity) to 20 points (full activity); mobility: 0 points (no mobility) to 40 points (full mobility); and strength: 0 points (0 kg) to 25 points (12.5 kg).

To measure postoperative satisfaction at the last follow up, the patients had to subjectively rate the results of the surgery with four possible answers: “very good,"(=1) “good,"(=2) “satisfied," (=3) or “unsatisfied" (=4). Pain was rated according to a Numeric analog scale (NAS) ranging from 0 (=severe pain) to 15 (=no pain).

### 2.3 Ethics and registration

All procedures carried out in studies involving human participants were in accordance with the ethical standards of the institutional and/or national research committee and with the 2013 Helsinki declaration and its later amendments or comparable ethical standards. The internal review board of the local university approved the study (project number S-305/2007).

### 2.4 Statistical methods

The data analysis was performed utilizing SPSS version 17.0 (SPSS Inc., Chicago, IL) and GraphPad Prism version 8.0 (GraphPad Software, San Diego, CA). Continuous variables were summarized with means and standard deviations. The Wilcoxon signed-rank test examined differences between preoperative and postoperative measurements. Within-group changes over time were analyzed using a one-sample *t*-test. Categorical data comparisons between groups utilized chi-squared tests. Kaplan-Meier survivorship analysis was conducted defining revision for any reason as the endpoint, specified as any operation with exchange of at least one component. P values less than 0.05 were considered statistically significant for all tests.

## 3 Results

**Fig. 1:**
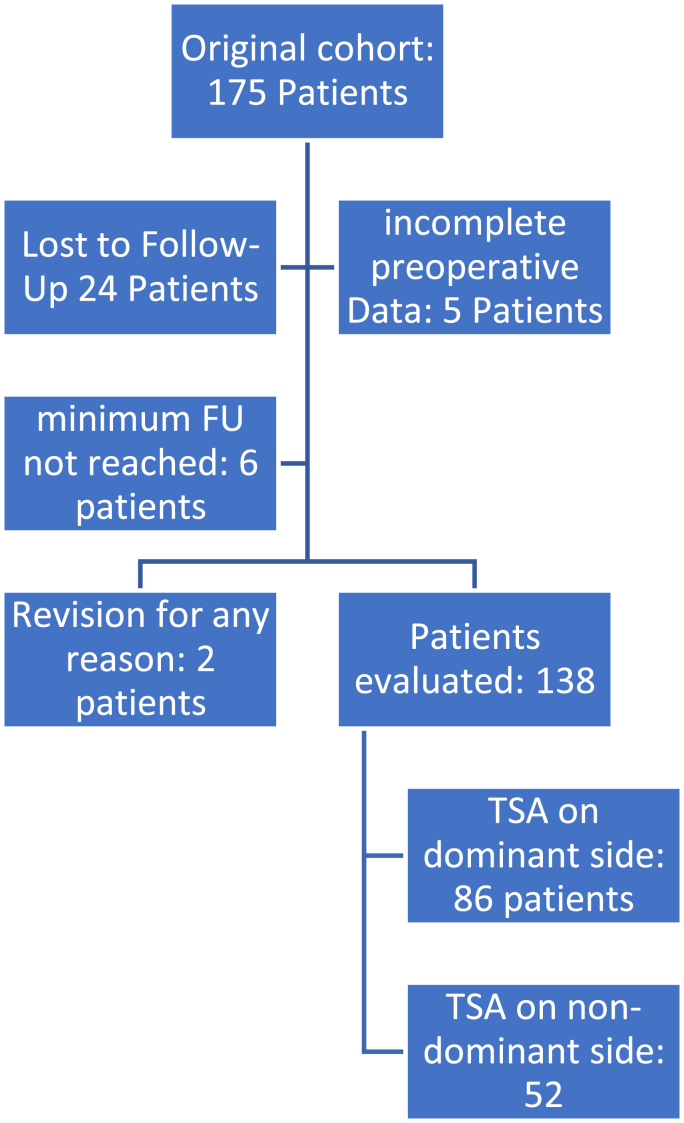
Overview of patient cohort.

24 patients were lost to follow-up and 5 had incomplete preoperative data, excluding them from analysis. Minimum follow-up of 24 months was required for inclusion, which 6 additional patients did not meet. Two patients underwent revision surgery for different reasons (one in each group). The remaining 138 patients were evaluated with a mean follow-up of 45 months (range 24–158 months) (Fig. 1). Mean follow-up was 44.3 months (range 24–123 months) in the dominant (DOM) group and 46.8 months (range 24–158 months) in the non-dominant (NON-DOM) group. Mean patient age at the time of surgery was 68.7 years (range 51–89 years). 104 patients (75.36 %) were women and 34 (24.63 %) were men. TSA was performed on the dominant side in 86 patients (62.32 %) (DOM group) and on the non-dominant side in 52 patients (37.68 %) (NON-DOM group). 54 patients (39.13 %) were left-handed and 84 (60.86 %) were right-handed.

### 3.1 Comparison of preoperative measurements with long-term follow-up

The mean follow-up in the DOM group was 44.3 months (Range 24–123 months) and 46.8 months (range 24–158 months) in the NON-DOM group. The mean age in the DOM Group was 68.2 years (range 51–85 years) and 69.5 years (range 54–85 years) in the NON-DOM Group. There was no significant difference in age between the groups (p = 0.389).

The mean CS improved significantly from 23.7 (range 6–54) preoperatively to 65.8 (range 21–85) in the non-dominant group and from 24.7 (range 5–50) preoperatively to 66.1 (range 10–88) in the dominant-hand group (p < 0.0001). The mean improvement in the non-dominant group was 42.1 (range 15–65) and 41.5 (range -10-80) in the dominant group. The difference in improvement between the two groups was not significant (p = 0.800).

The pain was rated according to a NAS improved significantly from 2.1 (range 0–8) preoperatively to 12.8 (range 5–15) in the non-dominant group and from 2.5 (range 0–12) preoperatively to 13.1 (range 2–15) in the dominant-hand group (p < 0.0001). The mean improvement in the non-dominant group was 10.7 (range 2–15) and 10.6 (range -1-17) in the dominant group. The difference in improvement between the two groups was not significant (p = 0.948).

The activity and mobility subscores of the Constant-Murley assessment demonstrated significant improvement from preoperative to postoperative measurements in both the non-dominant (NON-DOM) and dominant (DOM) groups (p < 0.0001). However, no significant between-group differences were detected for either activity (p = 0.894) or mobility (p = 0.922).

### 3.2 Subjective satisfaction rate

Postoperative subjective satisfaction was high among the 138 patients without revision surgery; 105 patients (76 %) rated their outcome as very good, while 22 (15.9 %) considered the results good. Only 11 patients (7.97 %) rated the result as “satisfied”or “unsatisfied” with the result. In the DOM group 91.86 % rated the result at least good or very good, in the NON-DOM group 92.31 % with no significant difference (p = 0.83).

### 3.3 Survival and complications

Two adverse events occurred. One non-dominant side patient underwent revision to reverse shoulder arthroplasty for aseptic loosening at 72 months postoperatively, but was subsequently lost to follow-up. A dominant side patient developed periprosthetic joint infection 13 months after the index procedure. This was managed with a two-stage revision protocol entailing prosthesis removal, antibiotic spacer implantation, a course of intravenous antibiotic therapy, and re-implantation of a total shoulder arthroplasty after 3 months. At final follow-up 11 years after the revision surgery, this patient reported excellent subjective satisfaction.

**Fig. 2:**
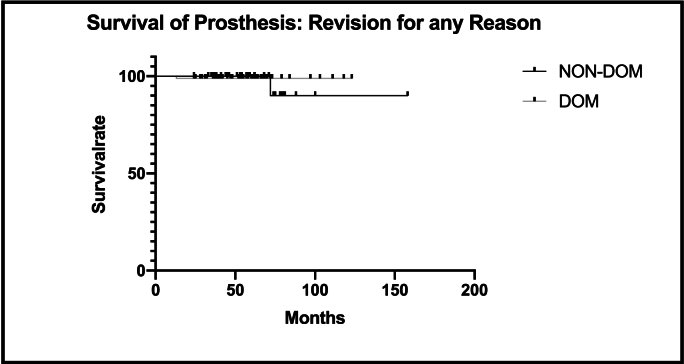
Kaplan–Meier survivorship for endpoint Revision for any Reason shows a survival of 98.8 % after 123 months in the DOM group and 90 % after 158 months in the NON-DOM group.

Kaplan-Meier survivorship analysis (revision surgery for any reason) demonstrated a survival of 98.8 % after 123 months for patients assigned to the DOM group and 90 % after 158 months within its respective NON-DOM group. No significant between-group differences detected (p = 0.89) (Fig. 2).

### 3.4 Functional results

The functional results for both the DOM and the NON-DOM group improved significantly from pre-to postoperatively in terms of range of motion (ROM). In the NON-DOM group preoperative Flexion (Flex) improved from 76.2° to 151.3°, abduction (ABD) from 65.8° to 141.0° and external rotation (ARO) from 9.9° to 42.7°. The power increased from 1.5 to 4.5. In the DOM group Flex improved from 81.6° to 149.2, ABD from 66.5° to 142.2° and ARO from 9.4° to 43.3°, the power increased from 2 to 5. The improvement of all values from preoperatively to postoperatively was significant in both groups (p < 0.0001), with no significant difference between the two groups (p > 0.1).

## 4 Discussion

Total shoulder arthroplasty has shown excellent medium-to long-term outcomes for the treatment of end-stage glenohumeral arthritis, consistently improving both pain and function.^1^, ^2^, ^3^, ^4^, ^5^ Nevertheless, several factors can influence subjective postoperative satisfaction, which has been widely investigated.^6^^,^^8^ The effect of hand dominance on postoperative range of motion, patient-reported outcomes, implant survival, and overall results remains controversial, with conflicting evidence in the literature.^14^, ^15^, ^16^, ^17^, ^18^, ^19^

In this cohort, there were no differences in age between the DOM and NON-DOM groups at the time of surgery, suggesting that hand dominance does not significantly influence the onset or progression of osteoarthritis in the shoulder joint.

This study found no significant differences in range of motion or patient-reported outcomes between the dominant and non-dominant sides following total shoulder arthroplasty. This contrasts with the findings of Cvetanovich et al., who reported greater postoperative elevation and external rotation following surgery on the dominant arm, although with shorter follow-up.^14^ It is possible that patients who undergo dominant-side surgery may experience a quicker clinical recovery after arthroplasty, potentially explaining greater motion during the first two years before reaching a final plateau.

Beyond range of motion, Cvetanovich et al. observed no significant differences in functional scores or pain between groups, which aligns with the results of the present study.^14^ Similarly, Berthold et al. also found no effect of hand dominance on range of motion or patient-reported outcomes.^23^

Several previous studies have reported that hand dominance does not predict clinical outcomes or implant survival following total shoulder arthroplasty.^17^^,^^18^ This finding is corroborated by our cohort, which showed no significant differences in prosthesis survivorship based on hand dominance during long-term follow-up.

Interestingly, rotator cuff tears have been reported at higher rates in dominant shoulders, likely due to increased mechanical load during use.^24^ However, this does not seem to adversely affect outcomes after total shoulder arthroplasty, as demonstrated by both the current study and multiple prior investigations.^14^^,^^23^^,^^25^

This study does have limitations, including the wide range of follow-up intervals and a relatively short minimum follow-up period of 2 years. Additional limitations include the balanced sample sizes between the two groups and the predominance of female patients in the cohort.

## 5 Conclusions

We found that neither the postoperative clinical results (ROM and PROMS) nor implant-survival have been influenced by hand dominance. Patients with primary OA in their dominant shoulder did not undergo surgery at an earlier age when compared to patients where the non-dominant shoulder has been affected. Therefore, patients can be counseled preoperatively that good-to excellent functional outcomes and implant survival after TSA can be expected regardless of their dexterity.

**Institutional Review Board Statement:** All procedures carried out in studies involving human participants were in accord-ance with the ethical standards of the institutional and/or national research committee and with the 2013 Helsinki declaration and its later amendments or comparable ethical standards. The internal review board of the local university approved the study (project number S-305/2007).

## Patient consent

Informed consent was obtained from all study participants.

## Conflicts of interest

“The authors declare no conflict of interest.”

## Author contributions

“Conceptualization, P.M. and P.H..; methodology, P.M. and P.H.; software, P.M. and J.D.; validation, P.M., D.S. and P.H.; formal analysis, P.H., T.B. and S.T.; investigation, M.W. and P.M..; resources, P.M..; data curation P.M. and P.H.; writing—original draft preparation, P.H.; writing—review and editing, all authors.; visualization, P.H. and J.D.; supervision, P.M. D.S.; project administration, P.M. and M.W.; fundingacquisition, P.M. All authors have read and agreed to the published version of the manuscript.”

## Declaration of generative AI and AI-assisted technologies in the writing process

During the preparation of this work the authors used generative AI - LLM for spellcheck and syntax in order to improve readability. After using this tool/service, the authors reviewed and edited the content as needed and take full responsibility for the content of the publication.

## Funding statement

No funding was received for the submitted work.

## Conflict of interest Statement

Pit Hetto: The author, their immediate family, and any research foundation with which they are affiliated have not received any financial payments or other benefits from any commercial entity related to the subject of this article.

Matthias Wolf: The author, their immediate family, and any research foundation with which they are affiliated have not received any financial payments or other benefits from any commercial entity related to the subject of this article.

Stefanos Tsitlakidis: The author, their immediate family, and any research foundation with which they are affiliated have not received any financial payments or other benefits from any commercial entity related to the subject of this article.

Julian Deisenhofer: The author, their immediate family, and any research foundation with which they are affiliated have not received any financial payments or other benefits from any commercial entity related to the subject of this article.

Thomas Bruckner: The author, their immediate family, and any research foundation with which they are affiliated have not received any financial payments or other benefits from any commercial entity related to the subject of this article.

David Spranz: The author, their immediate family, and any research foundation with which they are affiliated have not received any financial payments or other benefits from any commercial entity related to the subject of this article.

Paul Mick: The author, their immediate family, and any research foundation with which they are affiliated have not received any financial payments or other benefits from any commercial entity related to the subject of this article.
